# Circular vs. linear stapling after minimally invasive and robotic-assisted esophagectomy: a pooled analysis

**DOI:** 10.1007/s00423-022-02590-w

**Published:** 2022-06-22

**Authors:** Alida Finze, Johanna Betzler, Svetlana Hetjens, Christoph Reissfelder, Mirko Otto, Susanne Blank

**Affiliations:** Department of Surgery, Universitätsmedizin Mannheim, Medical Faculty Mannheim, Heidelberg University, Theodor-Kutzer-Ufer 1-3, 60167 Mannheim, Germany

**Keywords:** MIE, RAMIE, Esophageal anastomosis, Anastomotic leakage, Anastomotic stricture, Ivor-Lewis esophagectomy

## Abstract

**Purpose:**

Current data states that most likely there are differences in postoperative complications regarding linear and circular stapling in open esophagectomy. This, however, has not yet been summarized and overviewed for minimally invasive esophagectomy, which is being performed increasingly.

**Methods:**

A pooled analysis was conducted, including 4 publications comparing linear and circular stapling techniques in minimally invasive esophagectomy (MIE) and robotic-assisted minimally invasive esophagectomy (RAMIE). Primary endpoints were anastomotic leakage, pulmonary complications, and mean hospital stay.

**Results:**

Summarizing the 4 chosen publications, no difference in anastomotic insufficiency could be displayed (*p* = 0.34). Similar results were produced for postoperative pulmonary complications. Comparing circular stapling (CS) to linear stapling (LS) did not show a trend towards a favorable technique (*p* = 0.82). Some studies did not take learning curves into account. Postoperative anastomotic stricture was not specified to an extent that made a summary of the publications possible.

**Conclusions:**

In conclusion, data is not sufficient to provide a differentiated recommendation towards mechanical stapling techniques for individual patients undergoing MIE and RAMIE. Therefore, further RCTs are necessary for the identification of potential differences between LS and CS. At this point in research, we therefore suggest evading towards choosing a single anastomotic technique for each center. Momentarily, enduring the learning curve of the surgeon has the greatest evidence in reducing postoperative complication rates.

## Introduction

Esophageal cancer is still known for its rather poor prognosis with a 20% 5-year survival rate recently. Due to necessity of extensive therapy for curative treatment, especially postoperative complications and reduced quality of life are common [1].

Ivor-Lewis esophagectomy is described as the gold standard procedure in the current European Clinical Practice Guidelines of 2016. Although a laparoscopic abdominal approach is seen as standard procedure and unambiguously shows less and less severe postoperative complications in comparison to open esophagectomy [2], the thoracoscopic approach is not [3]. It is clear from today’s literature that minimally invasive esophagectomy (MIE), although usually associated with longer operative time, is associated to lower pulmonary complication rates as well as lower rates in cardiovascular complications [1, 4, 5].

Furthermore, postoperative pain, mean hospital stay, vocal cord paralysis, and mean estimated blood loss during surgery are significantly reduced in patients receiving MIE when compared to traditional open esophagectomy. Mean harvested lymph nodes and anastomotic leakage rate, however, do not differ between those two groups [5].

While MIE is not yet seen as a standard procedure but becoming increasingly popular, mechanical anastomosis is already performed routinely in esophagectomy [6]. Mechanical anastomoses can be performed using linear stapling (LS) and circular stapling (CS) devices. In open esophagectomy, data concludes that stapling techniques seem to be associated with lower rates of anastomotic leakage in comparison to hand-sewn anastomoses. This was shown in a metanalysis by Kamarajah et al. in 2020 which included mostly open esophagectomy and very few trials investigating MIE and robotic-assisted minimally invasive esophagectomy (RAMIE) [7]. Therefore, analyses towards minimally invasive surgery are still pending.

Since minimally invasive surgery has proven to show less complications and reduced mean hospital stay, study data including open surgical procedures can be applied to MIE or RAMIE only with caution and in a limited matter. A clear guideline towards usage of a specific mechanical anastomosis in MIE or RAMIE is currently not accessible.

Therefore, the Oesophago-Gastric Anastomosis Audit (OGAA) proposed to focus on the technical approach and surgical techniques in esophagectomy in January of 2021, putting special emphasis on research regarding anastomotic technique [8]. This is undermined further by a recent systematic review and metanalysis by Kamarajah et al., stating that anastomotic leakage has a negative prognostic impact on long-term survival [9]. This study aims to create an overview over direct comparison between circular and linear stapling in MIE and RAMIE concerning typical and major complications in patients receiving esophagectomy. As a result, an expert opinion on anastomotic technique in MIE and RAMIE will be conducted.

## Materials and methods

A pooled analysis of current literature on mechanic anastomosis types in direct comparison in MIE and RAMIE was created.

### Search strategy

A computer-based literature search was performed in several different databases, including the Cochrane Central Register of Controlled Trials (CENTRAL), the Cochrane Database of Systematic Reviews (CDSR) from The Cochrane Library, PubMed (NCBI platform 1966 to present), Cinahl (1981 to present) and Web of Science (1945 to present). No language restrictions were applied. The Cochrane Highly Sensitive Search Strategy for identifying randomized trials in MEDLINE, sensitivity maximizing version, were employed with predefined search terms to identify RCTs. It was adapted for the other databases searched. Moreover, the following online databases of ongoing trials were searched: www.clinicaltrials.nci.nih.gov.

The search was conducted in cooperation with a librarian and information specialist familiar with meta-analysis and lead by Cochrane Collaboration standards.

A full electronic search strategy for MEDLINE was conducted on Jan 29, 2021 (inception to search date): Number of hits: 338.

The following search terms were used: Circular stapl*, Linear stapl*, End-to-end, End-to-side, Side-to-side, Ivor-Lewis, esophagectomy, anastomotic leakage, delta-shaped anastomosis, triangulating stapling, esophageal carcinoma, anastomosis. synonyms for the search terms above were used. The hits include literature found by personal research.

Literature research was conducted again on January 17, 2022. No new data was found.

### Inclusion criteria

Inclusion criteria were set as the following.

1. Performed surgery had to be minimally invasive or robotically assisted
2. The anastomosis types described in the study hat to be exclusively mechanical and including linear as well as circular stapling techniques
3. Studies included needed to provide a direct comparison regarding typical complications and mean hospital stay between linear and circular stapling
4. Full text had to be available
5. The article had to be published in English
6. The articles needed to be published until Jan 29, 2021.

Conference abstracts, metanalyses, and systematic reviews were excluded.

### Data extraction

All the identified papers were screened by two individual researchers (SB and AF). All the publications meeting inclusion criteria were extracted and the full article was assessed. All double data were excluded.

### Definition of endpoints

Primary end points were defined to be typical complications and indirect measurement of patient recovery. These included anastomotic leak, anastomotic stricture, pulmonary complications, and mean hospital stay.

### Statistical analysis

For all calculations, the Review Manager version 5.3 (The Cochrane Collaboration, The Nordic Cochrane Centre, Copenhagen, Denmark) was used. Dichotomous data was analyzed using the inverse variance method and reported as odds ratio. Forest plots were used for visualization of the pooled results. The heterogeneity of studies was calculated using the *I*^2^ index. An *I*^2^ value of – 25% represents insignificant heterogeneity; > 25–50% low heterogeneity; > 50–75% moderate heterogeneity; and > 75% high heterogeneity[10]. Analysis with insignificant heterogeneity were calculated using a fixed-effects model and with a low or moderate heterogeneity using a random-effects model. A *p*-value of less than 0.05 was considered as statistically significant.

## Results

**Fig. 1:**
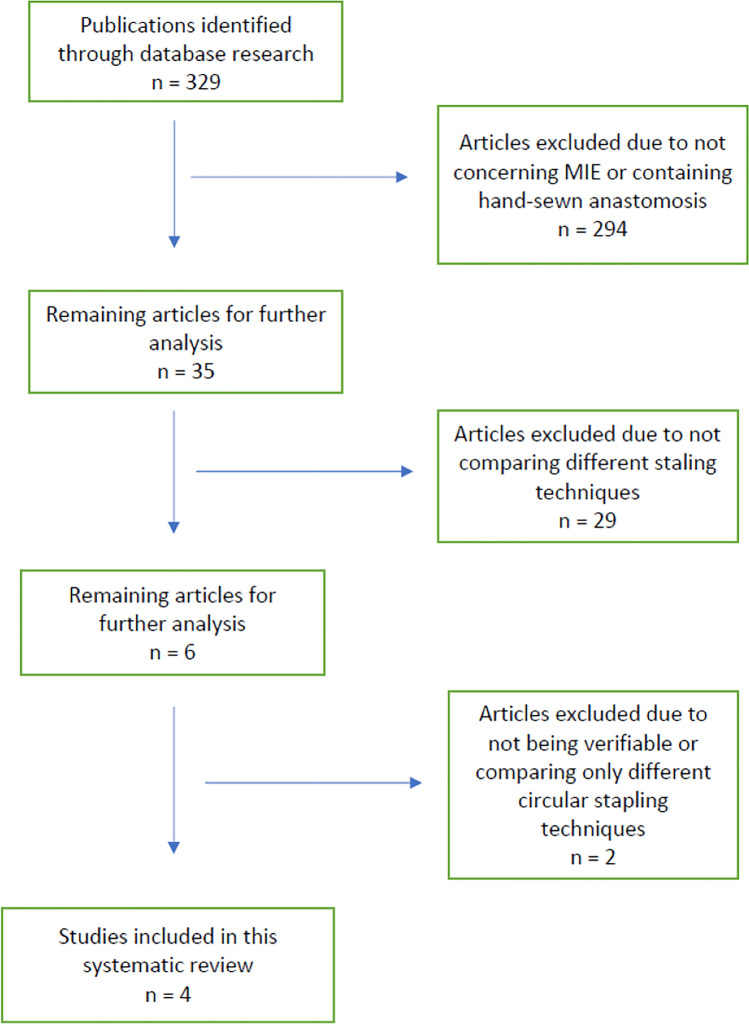
Comparison of mechanical anastomotic techniques in MIE and RAMIE. PRISMA flow chart

A total of 338 hits were generated through the search strategy above. Nine articles were identified as duplicates and removed from screening. A total of 329 articles were then screened. Two hundred ninety-four articles were identified not to meet the inclusion criteria concerning minimally invasive procedure or mechanical stapling technique. A total of 35 articles were then analyzed towards comparison between linear and circular stapling techniques. Of these articles, 6 articles could be extracted, of which one paper compared two different circular stapling techniques; for another publication, only an abstract was available, leaving a total of 4 publications included in this pooled analysis. This is shown in Fig. 1.

The included articles and associated data are summarized in Tables 1 and 2.

**Table 1:** Included articles and associated data. All the studies were retrospective, within linear and circular stapling, different staplers were used. Neoadjuvant therapy, change in surgeon and surgical procedure were not always specified

Author	Stapler used	Closing of anastomosis	Anastomosis height
Li (2014)	Circular: not further specified Linear for TS ATB 45-mm Ethicon	Stapled in linear Circular not specified	Cervical
Zhang(2019)	Circular: 25-mm stapler with 3.5-mm staples (CDH stapler, Ethicon Endo-Surgery), not further specified Linear: 3.8-mm staples not further specified	Linear: continuous single-layer barbed suture Stratafix Spiral 3/0 Circular: purse string stapling	Thoracic
Mungo(2016)	Linear: not specified Circular 3.5 and 4.8 mm 25 mm EEA + 25 mm Orvil	Not specified	Thoracic
Tian (2020)	TST: linear stapling not further specified TS: linear stapler not further specified Circular: not further specified	TST: linear TS: stapling Circular: purse string stapling	Cervical

**Table 2:** Within similar surgical techniques, stapling methods were not consistent

Author	Design	*N* linear	*N* circular	Anastomosis types	Surgical procedure	Neoadjuvant therapy	**Change in surgeon**
Li (2014)	Retrospective	33	51	Triangulating vs. circular stapled	Ivor-Lewis	Included	No
Zhang (2019)	Retrospective	35	42	Side-to side linear vs. end-to-side circular	Ivor-Lewis	Included	No
Mungo (2016)	Retrospective	12	38	3.5 mm EEA circular 2.8 mm EEA circular linear side-to-side	Ivor-Lewis	Included	Unknown
Tian (2020)	Retrospective	137	87	Circular unknown vs. triangulating vs. T-shaped anastomosis	Unknown	Excluded	No

**Fig. 2:**
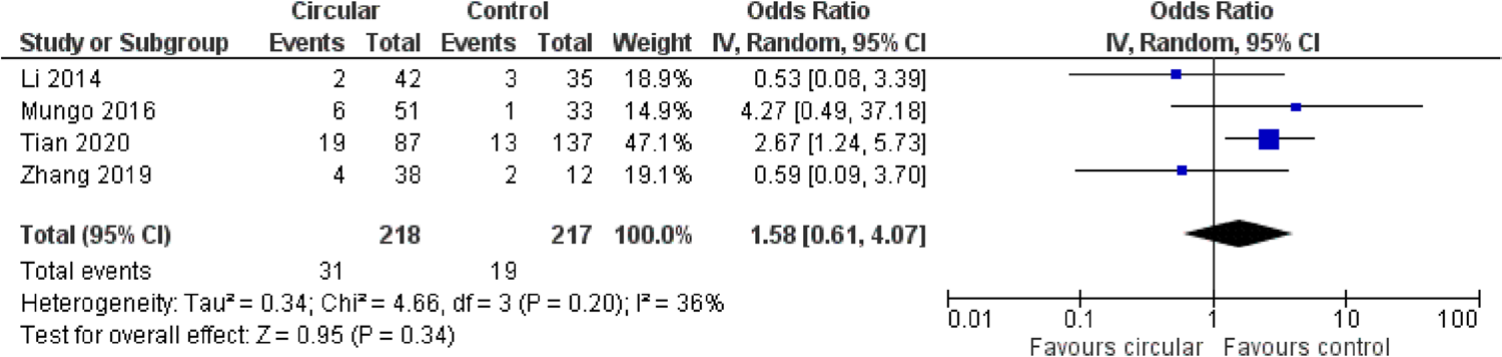
Odds ratio (OR) and combined analysis via forest plot (*p* = 0.34) show no favoring for a certain anastomotic technique regarding anastomotic leakage; OR is shown with 95% confidence interval (CI)

**Fig. 3:**
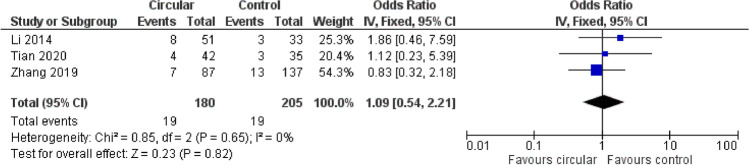
Odds ratio and combined analysis via forest plot (*p* = 0.82) shows no favoring for a certain anastomotic technique regarding pulmonary complications; OR is shown with 95% CI. *Pulmonary complications including pneumonia, pleural effusion, pleural empyema

A forest plot analysis (Figs. 2 and 3) showed no trends toward favoring either linear stapling technique or circular stapling technique.

The results of the individual articles are displayed in Table 3.

**Table 3:** Individual results for major complications and hospital stay. The included studies do not show differences in major complications between linear and circular stapling techniques in the documented cases (*p* > 0.05)

Author	CS Anastomotic leakage	LS Anastomotic leakage	*p*-value	CS pulmonary complications	LS pulmonary complications	*p*-value
Li (2014)	11.8%	3.0%	*p* = 0.31	15.7%	9.1%	*p* = 0.586
Zhang (2019)	4.8%	8.6%	*p* = 0.83	9.5%	8.6%	*p* = 1
Mungo (2016)	10.5%	16.5%	-	-	-	-
Tian (2020)	21.8%	9.5%	-	8.1%	9.5%	-
Author	CS mean hospital stay		LS mean hospital stay		*p*-value	
Li (2014)	10 d		10 d		*p* = 0.799	
Zhang (2019)	12.8 d		11.7 d		*p* = 0.37	
Mungo (2016)	9.7 d		10 d		-	
Tian (2020)	18.6 d		14.9 d		-	

Due to the small number of results, different LS anastomoses were included in the trial. Justification was drawn from literature in open esophagectomy.

### Anastomotic leakage

The publications by Li et al. and Zhang et al. showed no significant difference in anastomotic leakage with *p* being 0.31 and 0.83, respectively [11, 12]. Mungo et al. did not provide a *p*-value comparing any of the named complications as well as the mean hospital stay [13]. Tian et al., though, could show a clear difference in risks for anastomotic leakage between the tree displayed groups, including triangulating stapling (TST), T-shaped anastomosis (TS), and circular stapling (CS). With *p* = 0.29, the data states that patients receiving CS had significantly higher risk of developing anastomotic leakage than those treated with TS or TST via linear stapling [14].

Our forest plot analysis comparing all four included articles showed differing trends favoring circular stapling and linear stapling technique (*p* = 0.34) and, therefore, no overall significant difference between LS and CS.

### Pulmonary complications

Regarding pulmonary complications, our forest blot analysis showed similar results. Mungo et al. could not be included due to incomplete data; nevertheless, the analysis showed no overall significant difference in pulmonary complications (*p* = 0.82). Similar conclusions were made in all the three studies individually [11, 12, 14].

### Length of hospital stay

Due to missing data, a forest plot analysis could not be performed for the length of hospital stay. Nevertheless, Zhang et al. and Li et al. found no significant difference in length of hospital stay (see Table 2) [11, 12]. However, a significant difference in hospital stay (p = 0.000) was found in the study by Tian et al. Tian et al. differentiated between CS, TS, and TST with mean hospital stays of 18.63 days, 13.89 days, and 16.09 days, respectively.

### Further complications and parameters

The studies listed here concentrated on different interindividual complications, therefore limiting comparison between the studies.

Zhang et al. found no complication listed in their study (including hoarseness not specified towards recurrent laryngeal nerve palsy, dysphagia not specified to anastomotic stricture, chylothorax, and atrial fibrillation) to be significantly more likely in patients treated with CS or LS after esophagectomy. A single difference between the two groups was noted for anastomotic time (*p* < 0.01) stating that CS was significantly faster than LS.

Li et al. reported a significant difference in overall complications between patients undergoing MIE with CS and LS. In this case, the patients with LS anastomoses have shown a 15.2% complication rate only, while the patients with CS had an overall complication rate of 35.3% (*p* = 0.043). Specifically, the patients with CS seemed to develop more gastrointestinal complications not further specified than the patients with LS (25.5% and 3% respectively, *p* = 0,006). LS was performed after August 2013, while CS anastomosis was performed until July 2013 [11].

Tian et al. also found some further significant differences between the patients treated with CS, TS, and TST. Differences found were specifically for the need of intervention with pleural effusion (*p* = 0.037), TS requiring significantly more intervention when pleural effusion occurred. In addition, the patients receiving TST anastomosis were found to have a significantly lower risk for postoperative gastroesophageal reflux disease. Anastomosis time was lowest in TS, and operation time was lowest in TST (*p* = 0.000 for both) [14].

Overall, the pooled analysis performed could not find an overall significant difference in anastomotic leakage, pulmonary complications, and mean hospital stay when comparing CS to LS anastomoses in MIE and RAMIE.

### Further details

The included studies matched for the inclusion criteria above but did show some fundamental differences concerning surgical technique.

Details concerning staplers used, stapling technique, height of anastomosis, and surgical fastening of the anastomosis (specifically LS) are listed in Table 1.

It should be noted that none of the publications mentioned, whether intraoperative verification of adequate perfusion of the gastric tube was performed. Furthermore, it is not evident whether endoscopic vacuum therapy was performed with preventive intent in any of the patients.

Therefore, exact comparison of the data in the included studies is not possible.

## Discussion

Although an overall trend could not be identified in this pooled analysis, the individual studies did show some significant trends towards single complications.

Nevertheless, it should be considered that two of the included publications (Li et al. and Mungo et al.) described a change in stapling techniques over time with no overlap between usage of different anastomotic techniques [11, 13]. This leaves a possible bias through an effect of a learning curve.

Also, Tian et al. excluded all the patients with neoadjuvant radio- or radio-chemotherapy (RCT) from the trial [11], biasing the usual population undergoing esophagectomy. On the other hand, possible bias through previous radiotherapy was eliminated this way.

The included studies have further limitations concerning interindividual comparison when looking at details in surgical procedure. This is especially noticeable for the anastomotic technique and anastomotic height used. Schröder et al. demonstrated a clear connection between anastomosis type and anastomotic leakage rate (leakage rates being especially high in end-to-side double-stapling (23.3%) and cervical end-to-side hand-sewn (25.1%) in minimally invasive esophagectomy). Furthermore, when looking at the site of anastomosis, a clear difference in postoperative complication rates could be identified, favoring intrathoracic reconstruction over cervical reconstruction (*p* = 0.019) [15].

The included papers differ in height of anastomosis site and anastomosis type. This makes a comparison between the different trials as well as the inclusion of all the trials used in this pooled analysis together rather difficult. Interindividual comparison between different LS anastomoses is not always appropriate, similarly cervical anastomoses and thoracic anastomoses. There is some proof that cervical anastomoses might be associated with higher postoperative complication rates when compared to thoracic anastomoses [15, 16]. This might be responsible for some bias included in this trial.

Nevertheless, since there is so little data, it can be argued that if there were significant differences in postoperative complications due to anastomotic shape, these should appear in both cervical and thoracic anastomoses. Important details towards anastomotic technique including verification of adequate anastomotic perfusion were not mentioned by the authors, making comparison between the studies even more difficult. It should also be considered that cervical anastomoses are usually completed outside the situs and therefore labeled as minimally invasive; they do, however, formally contain an open portion of the surgical procedure.

A further possible bias regarding comparison of the papers included in this pooled analysis is the type of linear stapling technique used. The trial by Tian et al. found some differences regarding postoperative complications between triangulating stapling and T-shaped anastomosis. However, in addition to lack of data comparing different LS anastomoses, Tian et al. described a higher rate of anastomotic leakage and therefore prolonged hospital stay in CS when compared to all combined LS methods [14]. Currently, until more data is available, we therefore decided to combine linear stapling methods. In terms of practicability, combining LS methods is also relevant, since CS overall is becoming more popular due to being a simpler and easier technique than LS anastomoses, making an overall comparison of “other anastomotic techniques” and CS most relevant.

For circular stapling, however, choice of stapler and anvil size does not seem to matter significantly and should be adapted to a patient’s individual needs [17].

Regarding anastomotic stricture, data extracted from the publications was not specific enough as to allow a statement towards whether patients had verifiable anastomotic stenoses or solely symptoms of dysphagia.

Metanalyses in open esophagectomy have shown that linear stapling can reduce the risk of anastomotic failure, meaning anastomotic insufficiency, and depending on the technique, also the risk of anastomotic stricture when compared to hand-sewn (HS) anastomoses [18, 19]. However, this could not be reproduced in all the trials [20]. Similar results were displayed in a systematic review by Kamaranjah et al. concerning a comparison between CS, LS, and hand sewn anastomosis. The rate of anastomotic leakage was lower in the groups with LS and CS than in hand sewn anastomoses (*p* = 0.01 and *p* = 0.027, respectively). Furthermore, LS anastomoses had lower overall stricture rates than HS anastomoses (*p* < 0.001) [7].

A further metanalysis by Deng et al. from 2015 revealed less anastomotic strictures in LS and fewer anastomotic insufficiencies in cervical LS when compared to HS anastomoses [21].

Overall, in comparison to hand-sewn anastomoses, there seems to be at least some relevant degree of advantage regarding complications in mechanical anastomoses for open esophagectomy.

Furthermore, metanalyses for open esophagectomy have stated that the patients receiving LS anastomoses after esophagectomy tend to have fewer anastomotic strictures in comparison to the patients receiving CS anastomoses while having similar rates of anastomotic insufficiencies [22, 23]. Yanni et al. even displayed higher rates in anastomotic insufficiency in the patients receiving CS when compared to LS [24].

Although a metanalysis by Markar et al. in 2013 could not find a difference in anastomotic insufficiency when comparing minimally invasive esophagectomy to open esophagectomy [25], the insight that minimally invasive esophagectomy bears significantly lower risks for postoperative complications and in-hospital mortality [26, 27] as well as reducing postoperative pain is not new [27]. Long-term survival, however, does not seem to differ between MIE and open resection [28].

Considering the current known data for open esophagectomy, one could speculate that there might be differences between mechanical anastomotic techniques in MIE and RAMIE. However, current data is very sparse, as our pooled analysis shows.

This is especially interesting when regarding the fact that lower postoperative complication rates in MIE and RAMIE combined with the most beneficial type of mechanical anastomosis could offer the optimum therapy for the individual patient.

For the choice of mechanical anastomosis, technical difficulties, however, should not be excluded from consideration. A triangulating anastomosis possibly requires extended mobilization of the gastric tube and might therefore not be anatomically possible in every patient.

Nevertheless, since data does not give us a clear recommendation for which stapling technique to use, a standardized and therefore comparable procedure can make exchanging experiences easier. Decisions on which anastomotic technique to use need to be made, regardless of the lack of data. Egberts et al. have shown that standardizing RAMIE can be possible, as long as a consented decision is made on which anastomotic technique to use [29]. As mentioned above, the learning curve of each surgeon or center plays a very large role in outcomes of MIE and RAMIE. When comparing different stapling techniques during the stable phase of a learning curve, the results in postoperative outcomes and complications assimilate [30]. This plays an especially important role when considering that anastomotic leakage has a significant negative prognostic impact on long-term survival [9].

We therefore recommend choosing a single anastomotic technique for each center and to then endure the learning curve. Currently, we see this to be the most effective method for long-term reduction of complication rates.

The limitations of this study include the partially differing trial designs of the included studies, the exclusion of the learning curve as a possible bias in some trials, and the only partially overlapping documented postoperative complications. Furthermore, this pooled analysis only includes a very small number of trials, since data is limited.

## Conclusions

In conclusion, there is a lack of data concerning the comparison between CS and LS in MIE and RAMIE. To date, no real recommendation can be given based on the existing data.

Complementing the results of the OGAA, there is an urgent necessity for further randomized, controlled research regarding anastomotic technique in MIE and RAMIE.

Until deliberate recommendations can be given on anastomotic techniques, patient safety through high-volume centers needs to remain the main point of concern.

## Data Availability

As previously described in the “Materials and methods” of this article, the following databases were used: the Cochrane Central Register of Controlled Trials (CENTRAL), the Cochrane Database of Systematic Reviews (CDSR) from The Cochrane Library, PubMed (NCBI platform 1966 to present), Cinahl (1981 to present) and Web of Science (1945 to present).
