# Correction to: Circular vs. linear stapling after minimally invasive and robotic-assisted esophagectomy: a pooled analysis

**DOI:** 10.1007/s00423-022-02621-6

**Published:** 2022-07-25

**Authors:** Alida Finze, Johanna Betzler, Svetlana Hetjens, Christoph Reissfelder, Mirko Otto, Susanne Blank

**Affiliations:** Department of Surgery, Universitätsmedizin Mannheim, Medical Faculty Mannheim, Heidelberg University, Theodor-Kutzer-Ufer 1-3, 60167 Mannheim, Germany

**Correction to** **:** **Langenbeck’s Archives of Surgery**

10.1007/s00423-022-02590-w

The original version of this article unfortunately contained a small mistakes in the last names of the authors appear as first names.

The corrected author names are shown below:

Alida Finze · Johanna Betzler · Svetlana Hetjens · Christoph Reissfelder · Mirko Otto · Susanne Blank

The original article has been corrected.

